# A Preliminary Study to Explore the Informed Consent Approach and the Ethical Challenges in the Malaysian Biobanking for Research

**DOI:** 10.1007/s41649-022-00229-y

**Published:** 2022-11-08

**Authors:** Amnah Azahar, Aimi Nadia Mohd Yusof, Zahir Izuan Azhar

**Affiliations:** 1 Faculty of Medicine, Department of Medical Ethics and Law, Universiti Teknologi MARA, Sungai Buloh, Malaysia; 2 Faculty of Medicine, Department of Public Health Medicine, Universiti Teknologi MARA, Sungai Buloh, Malaysia

**Keywords:** Biobanking, Research ethics, Broad consent, Malaysia

## Abstract

Since 2005, Malaysia has established several biobanks to keep in line with the advancement of biomedical research and development of biobanks in other countries such as the UK and the USA. Despite the establishment of several biobanks in Malaysia, little is known about the informed consent approach in biobanking research and its ethical challenges. This study aims to identify the approach in obtaining informed consent in the Malaysian biobanking for research and explore its ethical challenges. Using non-probability purposive sampling, an in-depth interview with the key informants was conducted in Klang Valley. Based on the interviews, broad consent is the main approach used in obtaining informed consent in biobanking for research in Malaysia and five major ethical challenges were identified. These challenges include the informants’ opinion on the current informed consent approach, understanding participants’ rights, the role of the research ethics committee, biobanking governance in Malaysia, and informants’ knowledge and awareness. In summary, there is a lack of understanding among those involved in biobanking on the ethical, legal, and social aspects of biobanking for research in Malaysia.

## Introduction

The term “biobank” appeared for the first time in 1996 where Loft and Poulsen suggested a prospective biobank study to demonstrate the rate of oxidative DNA damage as an independent risk factor for cancer. In the Malaysian Guidelines on the Use of Human Biological Samples for Research, the term biobank is defined as a biorepository that receives, processes, preserves, and stores human biological samples and cells (National Committee for Clinical Research 2015). The samples stored in biobanks may contain associated data that includes genetic and personal information of participants (Elger and Caplan 2006). In biobank, samples are collected and stored for the use of current or future biomedical research that aims to improve the understanding of medical conditions including the diagnosis, progression, and prognosis, as well as the prevention and treatment of diseases (Smith and Aufox 2013).

In line with the advancement of biomedical research and the development of biobanks in other countries such as the UK and the USA, the Malaysian Government established its first population-based biobanking project in 2005 called The Malaysian Cohort (n.d.). Apart from The Malaysian Cohort, there are also institutional and private biobanks such as the University of Malaya Biobank (2021), Oral Cancer Research and Coordinating Centre (n.d.), Artificial Reproductive Clinics, Cryocord Sdn. Bhd., Cellsafe International (Malaysia) Sdn. Bhd., and StemLife Berhad (Abdul Aziz and Mohd Yusof 2019). Despite the promotion of benefits to healthcare in biobanking, the uncertainty within the practice creates many ethical challenges. One of the main ethical challenges lies with the process of obtaining informed consent from participants especially in meeting the criteria for a valid informed consent. A valid informed consent requires that a participant receives adequate information, being competent to understand the information given and able to provide the consent voluntarily (Beauchamp and Childress 2009). The problem with biobanking in relation to informed consent is the difficulty in providing adequate information for future research. It is possible that at the time when samples are collected from participants, the information on future research is still unknown (Shickle 2006; Macilotti 2013). Hence, the inability to provide specific information for future research at the time informed consent is obtained from participants. While it is acknowledged that valid informed consent cannot be achieved in biobanking, this paper recognizes the term ‘informed consent’ as it is nonetheless commonly used when consent in biobanking is discussed.

Although the National Committee for Clinical Research, Ministry of Health Malaysia has provided Malaysian Guidelines on the Use of Human Biological Samples for Research in 2015 and some literature has been published on the establishment of biobanks in Malaysia, little is known about the approach in obtaining informed consent from participants of these biobanks and the associated ethical challenges. This paper aims to identify the current approach in obtaining informed consent in Malaysian biobanking for research purposes and its ethical challenges.

## Methods

This study used a qualitative method as it allowed the description of individuals’ common meaning of experiences or concepts (Creswell and Poth 2018). By adopting this method, face-to-face in-depth interviews were conducted to understand the current approach of informed consent in Malaysian biobanking for research and to explore the informants’ knowledge and perception on the ethical challenges of the approach.

### Participants

A total of nine informants were selected randomly using non-probability purposive sampling that allowed maximum usage of the specific information related to the field. The informants who met the inclusion criteria as follows: (i) above 18 years old, (ii) involved in biobanking research, or (iii) familiar and knowledgeable with research in biobanking, were chosen from public or institutional biobanks, research ethics committees, and individuals with legal background. As this study focuses mainly on the approach of informed consent in Malaysian biobanking for research purposes, private biobanks were excluded in this study.

Informants who were chosen based on their involvement in biobanking research were those involved directly with research using biobanking samples. These include researchers, research assistants, and science officers. On the other hand, informants who were chosen based on their familiarity and knowledge of research in biobanking were not directly involved with research using biobanking samples but have worked academically in the area such as publishing work or worked with research governance bodies. The legal informants and members of the research ethics committee were categorized in this inclusion.

### Data Collection

Data collection took place in the Klang Valley from August 2019 until September 2019. Informed consent was obtained from all informants included in this study. The interview sessions were conducted in English and took place at the informants’ offices. Each interview session took about 20–30 min and was recorded using a voice recorder. All informants were interviewed using standard open-ended English questions. The following questions were asked during the interview:

- What is the consent approach in Malaysian biobanking for research purposes? Please explain the rationale of the approach.
- How does the consent approach protect participants’ autonomy or rights in the future?
- What are the ethical issues or challenges related to the consent approach?
- What are the possible legal issues that may arise related to the consent approach?
- How is biobanking being regulated in Malaysia?
- How does the law or regulation protect participants’ autonomy in future research?

### Data Analysis

The data were analyzed using a thematic analysis method which involved breaking down the transcripts into a manageable and meaningful text segments using a coding framework (Braun and Clarke 2006). The transcripts were read and coded into manageable and meaningful text segments by the first author (AA). Once all the transcripts have been coded, themes were developed based on the repetition and recurrence of the codings by the first and second authors (AA and ANMY). The third author (ZIA) helped in reviewing the themes and assisted in making sure that the developed themes were relevant.

Tables and mind-mapping were used along the analysis process to help in the organization of the findings. Mind mapping was developed based on the codings identified earlier in the process to help generate relevant themes to the context of this paper. The mind map helped to visualize the overall key points from the findings. This process allowed careful analysis and ensured that any identified codings were not missed in the development of the themes.

### Data Trustworthiness

A few steps proposed by Shenton (2004) were applied to ensure data trustworthiness. AA is a medical officer with experience working in the research management department. ANMY is a research ethics expert and has experience in qualitative research, and ZIA is a public health medicine specialist who is also an expert in qualitative data analysis. Data triangulation was achieved by the involvement of informants from various areas. Different backgrounds of the informants ensured that the information given was not biased. Recruited informants were researchers, science officers, members of the research ethics committee, and those with legal backgrounds. Informants were given the opportunity to refuse to participate by early email invitation and informed consent. The opportunities to refuse enhances honesty of the informants when contributing the data. Before the interviews started, informal ice-breaking sessions were conducted to build rapport between the investigator and informants. These sessions allowed the investigator to gain the informants’ trust and provide better understanding about the informants’ background.

## Results

The demographic characteristics of the informants were described in Tables 1, 2, and 3. From the in-depth interviews, the current informed consent approach in most of the Malaysian biobanking for research purposes is broad consent.

**Table 1:** Gender of the informants

Gender	*n* (%)
Male	3 (33.33%)
Female	6 (66.66%)
Total	9 (100%)

**Table 2:** Background of the informants

Informant background	*n* (%)
Research or science officer	4 (44.44%)
Members of research ethics committee	2 (22.22%)
Legal	3 (33.33%)
Total	9 (100%)
Involve in biobank research	4 (44.44%)
Familiar with biobank research	5 (55.55%)
Total	9 (100%)

**Table 3:** Informants exposure to research ethics training

Exposure to research ethics training	*n* (%)
Specific exposure to research ethics training	5 (55.55%)
Not exposed	4 (44.44%)
Total	9 (100%)

Several themes have emerged from the data to be considered as the ethical challenges associated with the current informed consent approach. These include informants’ opinion on the current informed consent approach, understanding of the rights of participants, the role of research ethics committees, governance for biobanking in Malaysia, and informants’ knowledge and awareness. These will be discussed in detail in the sections below. In discussing the verbatim quotations of the informants, we have anonymized the name to protect their true identities.

### Informants’ Opinion on Current Informed Consent Approach

Exploring informants’ opinions on the current informed consent approach will allow better understanding on the informants’ view and their perception towards the approach.

Two informants agreed that, by using broad consent, it could facilitate research in the future. One of them responded.

Sometimes we have a leftover sample that the researcher feels can be used in the future when there is a new development. With broad consent, we can use the samples without the need for re-consent. (Informant 2)

In addition, one informant responded that by using broad consent, the nature of the research will not be restricted.

Broad consent is where you can broadly tell the nature of research that will be carried out in the future. (Informant 3)

By adopting broad consent in biobanking research, the informants agreed that it is easier to use biobank samples for future research. They claimed that:

By using broad consent, it is easier to collect the samples to be used for future research or for academic purposes compared to disease-specific or research-specific consent. (Informant 5)

It is easier to use broad consent when collecting a sample as future research has not been determined during the initial consent taking. (Informant 6)

Nevertheless, some of the informants felt that although broad consent may help the researcher in the future due to its practicality, the consent approach may undermine and limit the participants’ autonomy.

The current model is not sufficient to protect the participants’ autonomy in the future because it is a one-time consent. (Informant 3)

Broad consent is not sufficient to protect the participants’ autonomy in the future. Usually, the consent mentions that participants do not have the rights towards their sample, and that already undermines their autonomy. (Informant 9)

On top of that, two informants also raised the issues regarding participants’ data safety and data quality when using broad consent:

Quality of data is questionable as one-time consent would mean there will not be interaction with the participant anymore, and we do not know if there are any changes that occur within the lifetime. How do we know that the participants are still alive or have a disease? (Informant 3)

There is a risk that the data collected can be identified although they have been coded and anonymized. (Informant 2)

Nevertheless, one informant strongly agreed that broad consent protects participants’ rights in the best way, as quoted:

I strongly agree that this approach will protect the participants’ rights as data will be encrypted and anonymized. We will make sure that the data will not leak, and confidentiality is our main priority. Only the project leader and IT manager have access to the data, but it is not easy to access. Researchers are allowed to use only analyzed data and need to access it at our place only. (Informant 4)

### Understanding of Participants’ Rights

In the context of medical research, respect for autonomy means allowing a person to consent to participate in research and voluntarily provide his bodily samples upon receiving adequate information about the research. It also requires the researcher to acknowledge the rights that the participants have when they participate in the research.

The informants were also being asked on what they think about the rights of participants in receiving information when using the current approach. Some of the informants felt that the information provided is sufficient to ensure that participants are informed and can give consent. They are quoted as saying:

I think the information mentioned in the informed consent is sufficient. If participants have questions, they are free to ask the researchers, and we will explain further. (Informant 4)

I think there are no issues as participants are aware that the samples taken will be used for future research. So far, we have never encountered any situation in which participants ask for more information on what research will be done in the future. (Informant 1)

Information provided in the information sheet seems to be sufficient and enough to make participants understand and give their consent. (Informant 6)

Apart from the right for information, informants were also asked about participants’ rights over their donated samples when using the current approach. There was an informant who agreed that participants should have the right over their donated samples:

The participants should have the right to their samples. I believe people have right over their body samples. The right is in terms of how they use your samples. I think we have the right to determine how our samples are being used. (Informant 3)

In addition to the right over donated samples, participants should also have the right on the benefit-sharing of the samples or data and be informed of the incidental findings:

If the research and commercialization lead towards the establishment or development of a very expensive treatment or device, I think whoever donates the samples to materialize the research should be given some benefits of sharing. (Informant 3)

About the incidental finding, it should be informed to the participants on the finding. (Informant 2)

Although two informants argue that participants should have rights over their data, another informant claimed that participants do not have any rights over the samples or data:

We mentioned in the informed consent that the participants do not have any right towards the data. (Informant 4)

### Role of the Research Ethics Committee

It is well understood that research involving human participants requires review from the research ethics committee to protect the participants from harm as well as to protect their autonomy. The majority of the informants agreed that it is the role of the research ethics committees to protect and oversee the conduct of biobanking research.

We have an ethics review. I think now it is enough to protect the participants’ autonomy as the research has to be reviewed by the research ethics committee. (Informant 3)

Tissues are not provided to researchers easily. Each research project must undergo ethical review and ethical approval by the research ethics committee before they are allowed to use the stored sample. The research ethics committee will ensure that the research will have a scientific impact and is ethical to be done without jeopardizing participants’ autonomy. (Informant 1)

### Biobanking Governance in Malaysia

In view that biobanking research involves the collection of human biological samples and its associated data for future use, it is important to explore the available ethical and regulatory frameworks that govern the conduct of such research. From the interviews, the majority of the informants claimed that there is no specific and proper guideline for biobanking in Malaysia.

At the moment, we don’t have a specific regulation that governs biobanking research. (Informant 3)

In Malaysia, I am not aware of any regulation or guideline regarding biobanking research. (Informant 6)

There is no regulation for biobanking. They just started with the consortium. (Informant 7)

Biobanking is not being regulated. (Informant 9)

Nonetheless, some of the informants claimed that most public biobanks in Malaysia follow the international guidelines and have also established their own guidelines or standard operating procedures.

We refer to the International Society for Biological and Environmental Repositories (ISBER) best practice for sample collection and repositories and our consent is based on Peter MacCallum Cancer Center which is already established. (Informant 1)

Currently, there is no regulation but, if the biobank is established by universities, it usually will be governed by the universities. (Informant 8)

We have our Standard Operational Procedure (SOP) on sample retrieval. (Informant 1)

Some of the informants gave their opinion on the Personal Data Protection Act (PDPA) that might have a role in biobanking research. However, the role is unclear.

Maybe PDPA will protect the data, but it covers private entities only and is not specific. (Informant 1)

PDPA might protect the data, but the person must consent to a purpose. In biobanking, the consent is not specific. (Informant 2)

Maybe PDPA can provide the protection, but I am not sure to what extent. (Informant 6)

### Informants’ Knowledge and Awareness

From the interview sessions, informants’ knowledge and awareness were explored on various aspects which include their knowledge and awareness on other types of informed consent in biobanking, common ethical challenges related to informed consent in biobanking, the legal impact of informed consent, and any legislation that can protect participants’ involvement in biobanking.

Besides the current approach of broad consent, the majority of the informants were not well exposed to the other types of informed consent in biobanking. Some of the responses are quoted as below:

I’m not sure about other types of consent used in biobanking. (Informant 1 and 6)

Sorry. I don’t know the other types of consent. (Informant 4)

Nevertheless, one informant addressed another type of informed consent that can be used in biobanking.

We should adopt another model of informed consent other than the broad one. For example, dynamic consent. (Informant 3)

Although all the informants were familiar with biobanking research and some of them were involved in the research field, not all informants had the knowledge or awareness on the common ethical issues related to informed consent in biobanking. For examples:

I think there are no ethical issues as participants are aware that their sample will be used for future research. (Informant 1)

So far, I never faced any ethical issues or challenges. (Informant 6)

There is no requirement for re-consent as current samples are from the adult population above 35 years old and from minors. (Informant 5)

Those who were aware of the ethical issues commented:

One of them is when the participants want to withdraw their donated sample in the future. (Informant 8)

The ethical issue includes trustworthiness regarding data and samples management. It gives social impact and harms the community if the data can be linked and leads to stigmatization of specific genetic diseases. (Informant 2)

Challenges may be seen with regard to what information to provide in the consent. (Informant 5)

The issue arises when the samples are used for commercialisation for future treatment. Can the owner of the samples claim any part of the treatment? Besides, what specific measure will be involved if, from the sample, they discover some diseases that the participants are likely to have? (Informant 9)

Usually, the issue will arise when the research involves financial gaining. However, the research ethics committee does not govern the aspect of financial gain. (Informant 7)

## Discussion

### The Understanding of Broad Consent

Although all the informants claimed the current informed consent approach in Malaysian biobanking for research is broad consent, this study argues that the informants may not understand the true meaning of broad consent. In this study, the informants’ opinions on broad consent appear to be more closely related to the definition of blanket consent. Blanket consent is a consent given freely for all types of research and data uses without the need of further permissions (Thompson and McNamee 2017). In this sense, participants have no control or say over how their donated samples and data will be used in the future.

Meanwhile, broad consent can be defined as consent given for an unspecified scope of future research with few content limitations and restrictions not to conflict with individual religion and moral values. (Grady et al. 2015). It has been argued that broad consent practice is designed with the intention of providing the participants with information on the governance structure which includes information on the risks and benefits that are inherent to the biobank governance in question (Boers et al. 2015). Therefore, broad consent can be considered as informed consent given for future research with additional oversight from the governing bodies (Sheehan 2011). Thus, it will also protect multi-level stakeholders’ interests including the participants’ interests. Although some of the informants agreed on the possibility of broad consent to undermine participants’ autonomy in the future, it has been argued that the ability of broad consent to include information on biobank governance is sufficient to help participants provide an autonomous decision, and thus, protect the participants’ autonomy and rights in the future (Boers et al. 2015). Nevertheless, broad consent should include sensitive issues that might interfere with the decision-making process (Spellecy 2015).

Since most of the informants believed that information provided in broad consent might be sufficient, it can be argued that it could be a false belief due to their understanding on the nature of broad consent especially because they had never encountered any situation in which the participants questioned about the nature of future research. Furthermore, some of the informants never had specific training on research ethics and hence the issue on the rights of participants for information was not seen as a problem. The result of this study is similar with the Malaysian stakeholders’ view on biobanking research as they view it as less risky, beneficial, and having few moral issues (Hashim et al. 2017). Therefore, it is necessary to further explore the views of all the stakeholders involved in biobanking research on their understanding of informed consent specifically on broad consent since it is perceived as the widely used of informed consent approach in Malaysian biobanking.

### Informants’ Background and Training

This study showed that the informants’ background might have influenced their belief on the rights that participants should have in biobanking research. The informants with legal backgrounds supported participants’ rights compared to those who do not have a legal background such as the researchers and science officers. This could be because those with a legal background have better understanding of the law compared to the others. Thus, they were able to relate more on the rights issues.

This study also demonstrates a significant difference between the informants who had exposure to research ethics training compared to those who were never exposed to research ethics training. Those who never had exposure to research ethics training have a lack of knowledge and awareness on the types of informed consent, ethical issues, and legal consequences. Despite the arguments on the types of informed consent and its ethical and legal aspects in biobanking (Kegley 2004; Khan et al. 2014; Mackenzie 2014), 44% of the informants in this study who were involved directly in biobanking research were unsure of other types of informed consent in biobanking and its ethical and legal consequences. This study showed that although the informants were involved directly in biobanking research, experience alone may not be sufficient to help them understand and be aware of the ethical and legal implications in biobanking.

Also observed in this study is the pattern that those who were aware of the different types of informed consent and its ethical and legal consequences were those familiar with the law and research ethics governance. It may be because they have specific exposure and training on research ethics throughout their career. This study also suggests that exposure to proper research ethics training like short courses or workshop series could help give a better understanding of the ethical and legal issues surrounding biobanking.

### Dependability on the Research Ethics Committee

Although it has been argued that research ethics committees may have failed to provide adequate review and have given less weight on the risks associated with participation in research (Savulescu 2001,2002), this study shows that most of the informants believed it is solely the responsibility of the research ethics committees to protect participants’ rights and autonomy in biobanking research. The informants justified their rationale by the ethics application process in which all new research must undergo ethics review before samples or data in the biobank can be used for research. However, with thousands of ethics applications and limited number of human resources, it can be argued that it is sometimes difficult to oversee all the applications at one time. In addition, it has also been argued previously that the research ethics committee might sometimes be too eager to promote new research and might sometimes jeopardize participants’ autonomy (Hofmann 2009). Therefore, the findings of this study highlight the importance of having adequate knowledge and understanding on research ethics. As such, the issues related to participants’ rights and autonomy in biobanking research could be addressed early during the development of research protocol without heavily depending on the research ethics committees.

### Local Guidelines and Regulations on Malaysian Biobanking

This study found that majority of the informants were not aware of the existence of the Malaysian Guideline on the use of human biological specimens for research published by the National Committee for Clinical Research in 2015. This might probably be due to the inadequate information provided in the guideline to fully govern the conduct of biobanking research. This study also found that the conduct of biobanking research might be different among the biobanks as some of the biobanks followed international guidelines such as ISBER for sample repositories and some have established their own. As a result, regulating biobanking research can be challenging. As shown in a previous study by Hawkins and O'Doherty (2010), to earn participants’ trust in the biobank, it requires good governance via its transparency, accountability, and control mechanism. In addition, a study by Hashim et al. (2017) found that Malaysian stakeholders expressed high concern with issues of data and specimens’ protection.

Although not specific to biobanking, this study highlights the relevance of the Personal Data Protection Act in protecting sensitive data related to biobanking. Therefore, it is now clear that the country needs national guidelines and regulations specific to the conduct of biobanking that can address all the technical, operational, ethical, and legal concerns related to biobanking.

### Limitation of this Study and Future Research

Due to the limited number of experts in the field and the limited time to conduct this research, the number of informants had been limited to nine persons. The field of biobanking in Malaysia is still relatively new and we hope that future research would be able to expand the findings from this research and help provide guidance for researchers in obtaining informed consent in an ideal way that protects the rights and autonomy of research participants in biobanking.

## Conclusion

Broad consent is mainly used in the current informed consent approach in Malaysian biobanking and comes with many ethical challenges, especially in issues dealing with respecting participants’ autonomy. Although “The Malaysian Guidelines on the Use of Human Biological Samples for Research” published by the National Committee for Clinical Research, Ministry of Health Malaysia in 2015 is available to assist research, it is insufficient in providing guidance on the informed consent process. Hence, the recommendation to improve the local guideline is imperative to address the ethical challenges with the current informed consent approach.

Despite the establishment of a few biobanks in Malaysia, this study demonstrates the lack of knowledge and awareness associated with ethical issues and challenges in biobanking among those involved in the research. It is probably because of the lack of exposure to specific research ethics training among them. This study also showed that the majority of those involved in biobanking depend on the research ethics committee to govern the biobanking research and protect participants’ autonomy.

Perhaps, a more systematic research ethics training should be developed to be offered to all involved in research involving human participants. As for now, research ethics training depends on the initiatives of institutions. At the same time no available national syllabus on research ethics leading to variations in training. With the inadequacies identified, further studies in Malaysia on the area of informed consent in biobanking are needed to help develop a proper guidance for the informed consent process to uphold the rights of individuals who participate in biobanking research. Improvement in the research ethics governance is very much needed to ensure research involving human participants in Malaysia is conducted ethically.
